# The long walk to universal health coverage: patterns of inequities in the use of primary healthcare services in Enugu, Southeast Nigeria

**DOI:** 10.1186/1472-6963-14-132

**Published:** 2014-03-21

**Authors:** Ijeoma L Okoronkwo, Obinna E Onwujekwe, Francis O Ani

**Affiliations:** 1 Department of Health Administration and Management, University of Nigeria, Enugu Campus, Enugu, Enugu State, Nigeria; 2 Department of Nursing Sciences, College of Medicine, University of Nigeria, Enugu Campus, Enugu, Enugu State, Nigeria; 3 School of Nursing, Enugu State University Teaching Hospital, Enugu, Enugu State, Nigeria

**Keywords:** Utilization, Primary healthcare (PHC) services, Inequity, Universal health coverage (UHC)

## Abstract

**Background:**

Knowledge and understanding of health service usage are necessary for health resource allocation, planning and monitoring the achievement of universal coverage (UHC). There is limited information on patterns of utilization among adult users of primary health care (PHC) services. Lack of understanding of current and past utilization patterns of health services often hinders the improvement of future Primary Health Care (PHC) delivery in the remote areas of developing countries. This paper presents new knowledge on the patterns of utilization of PHC services among adults in Enugu metropolis southeast Nigeria.

**Methods:**

A cross-sectional study was conducted in 15 PHC facilities of Enugu North Local Government Area (LGA) from June to July 2012. A total of 360 consenting adult users aged 18 years and above were consecutively recruited as they attended the health facilities. An interviewer-administered questionnaire was used to collect data from the respondents. A modified Likert scale questionnaire was used to analyze data on patterns of utilization. Utilization of PHC services was compared by gender, socio-economic status (SES) and level of education.

**Results:**

Out of the 360 respondents, (46.9%) utilized PHC services regularly. The components of PHC mostly utilized by respondents were immunization with a mean score of 3.05, treatment of common ailments (2.99) and maternal and child health (2.64). The least poor SES group utilized PHC services the most while the very poor and poor SES groups used PHC services least. There were statistically significant relationships between utilization of PHC services and gender (p = 0.0084), level of education (p = 0.0366) and income (p = 0.0001).

**Conclusions:**

Most adult users in this study did not utilize the health facilities regularly and there were gender, educational and SES inequities in the use of PHC services. These inequities will negate the achievement of universal health coverage with PHC services and should be remedied using appropriate interventions.

## Background

The achievement of universal health coverage (UHC) is hinged on universal access to health services and financial risk protection for all people. However, the use of health care services varies among individuals, families and communities according to their needs, socioeconomic status, beliefs, values and expectations [1]. These factors determine where, when and how services are utilized with consequences on UHC [2].

Knowledge and understanding of health service usage are necessary for health resource allocation, planning, tracking the achievement of UHC and ensuring that informed decisions are made on how to improve access and achieve UHC [3]. Unfortunately, health service planning and policy decisions are often made without any clear understanding as to the characteristics of current utilization particularly in rural parts of the developing world, where few studies have been undertaken [3].

Lack of understanding of current and past utilization patterns of health services often hinders the improvement of Primary Health Care (PHC) delivery in the remote areas of developing countries and retards the achievement of UHC. However, patterns of utilization of health care services can be measured based on frequency of use for those making at least one visit to a health facility and identification of predictors of the number of subsequent visits [4].

Utilization of PHC services significantly contributes to determining the health outcomes of the community. It has been observed that when people are dissatisfied with the PHC services provided, they commute long distances from their villages to seek medical care at better equipped and staffed hospitals located in large towns [5]. Low levels of utilization of PHC services in Nigeria, especially in Enugu state, where the study was conducted have been reported [6]. An average of 3,063 clients representing about 25% of 12,253 clients attended PHC facilities in 2009 and utilized various PHC facilities in the Enugu metropolis health districts [6]. However, existing data appear not to contain adequate information on patterns of usage. Secondly little is known about the relationship between socio-demographic factors and patterns of usage of PHC services. The question often asked in most surveys concerns type of health facilities utilized and not patterns of usage [“unpublished data”].

This paper presents new information about the level of inequities in the patterns of utilization of Primary Health Care services in Enugu state, southeast Nigeria as a measure of the march of the state towards achieving UHC. It also elaborates on the interventions that can be used to improve equitable and universal access and use of PHC services. Specifically the paper shows the frequency of use of PHC services based on demographic and socioeconomic status.

## Methods

### Study design and setting

A cross-sectional study was conducted from June to July 2012 in Enugu North Local government area (LGA) of Enugu state, Southeast Nigeria. It is both a residential and commercial area and has both urban and rural dwellers. The LGA is made up of civil servants, traders and farmers. Despite efforts made over the years by the government towards human resource development and basic health infrastructure, the health system of the state has continued to suffer substantial problems [6].

### Participants

A simple random sampling technique was used to select Enugu North LGA out of the three LGAs in Enugu health district. An adequate minimum sample size of 385 was computed based on 95% confident interval and power of 80%. All adult males and females aged 18 years and above attending the health facilities were eligible to participate. The sample size was distributed based on proportionate size allocation of clinic attendance. Total monthly attendance prior to the commencement of data collection in each of the 15 health facilities was used for the allocation. This was obtained from the registers of the health facilities. A simple random sampling was used to select the respondents from the attendance register (sampling frame).

### Data collection

An interviewer-administered questionnaire was used to collect data. The questionnaire consisted of two parts. Part 1 comprised the respondents’ socio demographic characteristics. Part 2 was made up of two sections. Section A was made up of modified Likert Scale questions which was used to assess respondents’ opinion on patterns of utilization of PHC services while Section B comprised information on the occupation and specific household assets of respondents to determine their socio-economic status. Altogether there were 13 item questions. The questionnaire was translated into the main local language spoken in the area (Igbo) to enable clients that speak and understand Igbo complete the instrument. The instrument was pilot tested for reliability by administering 30 copies to adult PHC facility users in Enugu South health district with similar characteristics. The percentage scores of responses for each section of the instrument were analyzed using Pearson Product Moment Correlation Coefficient and Spearman Brown Prophecy formula for correlation score at 0.05 level of significance. The result for Section A (personal profile) was 0.8 while Sections B (Patterns of utilization) and C (household assets) were 0.8 and 0.82 respectively showing a high degree of internal consistency of the instrument. Patterns of utilization as used in this study refer to the use of some components of PHC which include health education, maternal and child health (MCH), immunization, treatment of common ailments, mental and dental health services.

### Ethics statement

The approval for the study was obtained from the Enugu Metropolis Health District Board Ethics Committee (Reference ENS/DHS/EMDHB/01/10). Informed consent was obtained from all the respondents, who were all given the option of not participating in the study if they so wished.

### Data analysis

Descriptive statistics was used for general description of study participants. The modified Likert scale was used to analyze patterns of utilization and scores were rated as: Strongly Agree (SA) = 4 points; Agree (A) = 3 points; Disagree (D) = 2 points and Strongly Disagree (SD) = 1 point. Average score was 2.5, therefore 2.5 and above was considered positive score. Principal components analysis (PCA) in STATA software package [7] was used to create continuous socio-economic status (SES) index [8,9] using information from the households’ asset holdings together with information from occupation. The assets were ownership of motorcar, motorcycle, radio, refrigerator, television set and bicycle. Standard assets were used in the study for both urban and rural dwellers so as to objectively and uniformly assess the SES of the households. The use of standard assets is most discriminatory for measuring SES [8,10]. Hence, whilst some assets are expectedly more prevalent in the urban area, some are more prevalent in the rural area. Income was not used in defining SES because people in Nigeria do not provide reliable information about their income. The first principal component of the PCA was used to derive weights (eigenvectors) for the SES index [11] and the mean, standard deviation and factor scores were computed. The positive factor scores and their magnitude indicated the degree of differences regarding the demographic variables in both urban and rural areas. The index was used to divide the respondents into four equal sized SES groups (quartiles).The quartiles were Q1 (most poor); Q2 (very poor); Q3 (poor); Q4 (least poor) SES quartiles. The frequency distributions of the variables by SES and rural–urban location were calculated and chi-squared (Chi^2^) tests for trend analysis were used to examine statistical differences across socioeconomic groups in utilization of PHC services at 0.05 level of significance.

## Results

### Demographic characteristics of respondents

The number of completed questionnaires available for data analysis was 360. The result in Table 1 shows that the lower age limit was 18 years while the upper age limit of the respondents was 53 yrs. The mean age was 34 yrs and standard deviation (SD) 8.73. Most of the respondents were females 259 (71.9%). More than half were married 199 (55.3%).

**Table 1:** Demographic characteristics of the respondents n = 360

**Demographic characteristics**		**f**	**%**
Age last birthday(in years)	18-23	46	12.8
	24-29	78	21.7
	30-35	101	28.1
	36-41	48	13.3
	42-47	63	17.5
	48-53	24	6.67
	-Mean age (x)	34 yrs	
	-Std dev. (SD)	8.73	
	-Variance (S^2^)	76.25	
Sex	Male	101	28.1
	Female	259	71.9
Level of education	No formal	45	12.5
	Primary	41	11.39
	Secondary	87	24.17
	Tertiary	187	51.94
Marital status	Single	135	37.5
	Married	199	55.3
	Widow	12	3.33
	Widower	10	2.78
	Divorced	4	1.11

### PHC services utilized by respondents

The PHC services most commonly utilized as identified by the respondents were immunization with a mean score of (3.05), treatment of common ailments (2.99) and maternal and child health care services (2.64) as shown in Table 2. The least utilized service was mental health services (2.05).

**Table 2:** PHC services utilized

**S/N**	**Item**	**SA**	**A**	**D**	**SD**	**X**
	I utilize the following PHC services in my health facility:					
a	-Maternal and child health care services	80	140	72	68	2.64
b	-Treatment of common ailments	100	190	36	34	2.99
c	-Health education	33	49	198	80	2.10
d	-Immunization	64	264	17	15	3.05
e	-Mental health services	14	30	276	40	2.05
f	-Dental health services	32	41	243	44	2.17

### Regularity in the use of PHC services

Out of the 360 respondents, 169 (46.9%) utilized the health facilities regularly, while 191 (53.1%) did not.

### Frequency of monthly utilization of PHC services

Out of the 169 respondents that utilized PHC services regularly, 45(26.6%) attended once per month, 68(40.2%) twice per month, 36(21.3%) utilized the services three times while 20(11.8%) used it four times and above per month.

### Reasons for not utilizing the facilities regularly

Table 3 shows the various reasons given by the respondents for not utilizing the health facilities regularly. They include: absence of health worker 57(29.8%); it is not necessary (ignorance) 37(19.4%); spouse disapproval 28(14.7%); poor service hours 26(13.4%); poor attitude of health workers 22(11.5%); and PHC services coincide with market days 21(11.0%).

**Table 3:** Reasons for not utilizing the facilities regularly n = 191

**Reasons**	**f**	**%**
Absence of health workers	57	29.8
It is not necessary (ignorance)	37	19.4
Spouse disapproval	28	14.7
Poor service hours	26	13. 6
Or attitude of health workers	22	11.5
PHC services coincides with market days	21	11.0

### Differences in respondents’ utilization of PHC services based on their demographic and socio-economic status

Table 4 shows that the least poor SES groups utilized PHC services most while the least utilization was amongst the most poor and very poor SES, male urban, male rural and female rural groups. The poor/rich (Q1/Q4) quartile ratio shows the level of gap that has to be bridged in order to ensure equity and improve the condition of the poorest households.

**Table 4:** Differences in respondents’ utilization of PHC services based on their demographic and socioeconomic status

**Variable description n (%)**		**Urban**		**Rural**	
		**Male (65)**	**Female (143)**	**Male (36)**	**Female (116)**
Highest educational qualification	No formal	9(13.8)	4(2.8)	17(47.2)	15(12.9)
	Primary	9(13.8)	10(7.0)	10(27.8)	12(10.3)
	Secondary	20(30.8)	35(24.5)	7(19.4)	25(21.6)
	Tertiary	40(61.5)	87(60.8)	14(38.9)	46(39.7)
Occupation	Unemployed	38(58.5)	60(42.0)	14 (38.9)	45 (38.8)
	Student/trainee	55(84.6)	50(35.0)	30(83.3)	45(38.8)
	Farmer	30(46.2)	15(10.5)	25(69.4)	35(30.2)
	Artisan	40( 61.5)	84(58.7)	30(83.3)	95(81.9)
	Small scale business (E.g., Petty trading)	65(100)	105(73.4)	25(69.4)	69(59.5)
	Medium/ large scale business	45(69.2)	45(31.5)	25(69.4)	39(33.7)
	Teacher	40(61.5)	115(80.4)	30(83.3)	100(86.2)
	Civil servant	50(76.9)	135(94.4)	20(55.6)	100(86.2)
Functional assets owned	Radio	63(96.9)	133(93)	36(100)	116(100)
	Television	65(100)	140(97.9)	30(83.3)	67(57.8)
	Bicycle	40(61.5)	35(24.5)	25(69.4)	56(48.3)
	Air conditioner	60(92.3)	138(96.5)	30(83.3)	57(49.1)
	Car	58(89.2)	100(69.9)	28(77.8)	22(19.0)
	Motor cycle	30(46.2)	30(21.0)	30(83.3)	30(25.9)
	Refrigerator	46(70.8)	136(95.1)	36(100)	93(80.2)
	Rechargeable lamp	60(92.3)	100(69.9)	30(83.3)	60(51.7)
	Electric fan	64(98.5)	134(93.7)	28(77.8)	34(29.3)
	Kerosene lamp	64(98.5)	138(96.5)	36(100)	107(92.2)
Socioeconomic status (in quartiles)	Q_1 _(most poor)	16(24.6)	36(25.1)	9(25)	29(25)
	Q_2 _(very poor)	16(24.6)	35 (24.5)	9(25)	29(25)
	Q_3_ (poor)	16(24.6)	35 (24.5)	9(25)	29(25)
	Q_4 _(least poor)	17(26.2)	37 (25.9)	9(25)	29(25)
	**Total**	**65(100)**	**143(100)**	**36(100)**	**116(100)**
	**Q1/Q4 (poor-rich ratio)**	**0.94**	**0.97**	**1.0**	**1.0**

Table 5 shows significant relationships between sex, educational status and use of PHC services using the chi-squared test at 0.05 level of significance.

**Table 5:** Relationship between sex and educational status with PHC services utilization

**Socio-demographic variables**		**Yes**	**No**	**Total**	**p value**
**Sex**	Male	47	54	101	0.0084*
	Female	160	99	259	
	Total	207	153	360	
	***x***^***2 ***^**6.94**				
**Levels of education**	Non-formal education	17	28	45	0.0366*
	primary education	27	14	41	
	secondary education	50	37	87	
	tertiary education	111	76	187	
	Total	205	155	360	
	***x***^***2 ***^**8.51**				

*p-value significant at 0.05 level.

## Discussion

Majority of adult users in this study were females. This is not surprising as the kind of services being offered at the PHC facilities e.g. antenatal, family planning, maternal and child health, immunization are more female oriented. This finding is in support with the study on utilization of primary health care services in the Tshwane region of South Africa [12] where more females 160(56.2%) than males 75(26.33%) utilized the services. The study also showed that only (46.9%) of the respondents utilized the PHC services regularly and major reasons for not using the PHC services regularly as indicated by the respondents include absence of health workers, ignorance and spouse disapproval. Our findings are similar to those in a study carried out in Oji River, Enugu state where reasons for non or under utilization of health facilities were absence of a medical doctor (84.1%), poor staff attitude (47.6%) and long distance (39.6%) [13]. Another similar study in Zaria, Kaduna state showed that (42.9%) of the participants were of the opinion that utilizing ANC services was unnecessary, while 36.1% attributed non utilization to spouse refusal [14]. Other reasons cited in this study include lateness to duty, early clinic closure and poor attitude of health workers. The behaviour of health workers can influence use of health facilities. Achievement of UHC may be a mirage under this situation. This calls for better orientation and communication between health care providers and clients.

The three PHC services most commonly utilized were immunization, treatment of common ailments, and MCH. This is similar to what was found in a study at Oji River Enugu state where utilization of immunization services, antenatal and delivery services were on the increase [13]. This may be due to the state government’s emphasis on MCH, with routine immunization services which are readily available in the health districts.

The poor utilization of mental health services reported in this study is similar to what was found in Israel where only 20% of the total population utilized mental health services in their life time [15]. Low utilization of health education, mental and dental health services as seen in this study could imply that the services were not readily available or that there was a low demand for them. Our findings are similar to what was reported in the study to determine the utilization of dental services in a population of Nigerian university students where there was low utilization of dental services; only 14% of the subjects utilized the services in the past one year. The reason given for poor utilization was that there was nothing wrong to necessitate a visit [16].

Our study showed that females utilized the PHC services more than their male counterparts in both urban and rural areas of the health district studied. Our findings are similar to what was reported in Central Asia on gender gap in PHC resource utilization [17] where women and children were the principal users of PHC in both urban and rural areas constituting over 80% of total visits to PHC while adult males had fewer than 25% of all visits in urban and 11.8% of visits in rural area. This may imply that females have more health care needs than males in this health district.

The findings also showed that there were significant differences between SES, educational attainment and utilization of PHC services. The least poor SES group (Q_4_) and those with higher educational attainment, utilized the PHC services most in the health district while the very poor and poor (Q_2_ & Q_3_) groups utilized services the least. PHC services are preventive services that should be geographically and financially accessible to all. The disparity in the use of PHC services across socio- economic groups is an area of concern among policy makers. Our findings are similar to those in the study on economic burden of malaria illness in south east Nigeria where the pattern of care seeking among the socio-economic groups indicated that the wealthier population groups have a higher probability of seeking care at the health centres [18]. The researchers further stated that “health interventions initially reach those of higher socioeconomic status and then, the poor”.

Education exerted a significant influence on the use of PHC services in this study. Many studies in other developing countries have found that education is one of the most important determinants in the utilization of health care services [19,20]. The reason could be that educated people may access information on health through the internet, or books and are more likely to utilize health services more than the uneducated. Education helps in developing confidence to make informed decisions regarding one’s health. This suggests that improving educational opportunities to adult users may have a large impact on the utilization of PHC services. However, our finding contrasts the study on maternal health utilization in which there was no difference in patterns of utilization among the educated and uneducated women [21].

It has been reported that geographic location appears to have an effect on utilization of services [22]. In this study, females who resided in urban areas utilized PHC services more than those in the rural areas. This urban/rural contrast is an indicator of the disparity that exists by place of residence in the utilization of PHC services in the area of study. This may be due to the fact that people living in urban areas have better road networks and transportation making it easier for them to have access to health facilities than those in rural areas; moreover women in urban areas are better informed than their rural counterparts.

The study has some limitations. It was confined to Enugu health district; therefore the findings cannot be generalized to all the PHC facilities in the other six health districts of Enugu state or the whole country. Furthermore the study utilized only those attending the facilities thereby excluding majority of the households within the community. Future research on patterns of utilization should be extended to other health districts using household survey. There is also the need to determine constraints to UHC in the utilization of primary health care services among socio economic groups in the health districts.

## Conclusions

In conclusion, the pattern of utilization of PHC services in the Enugu health district is unsatisfactory and implies that the health system surely has a very long walk to take before significant progress towards achieving UHC is made. There are significant differentials in utilization of PHC services such as educational level, urban/rural residence, gender and socio-economic status. To achieve equitable UHC, the right to access to health must be achieved across the society so that those who need care are able to access it regardless of who or where they live or their ability to pay. The poorest population is in greater need of health services than the richest population and should therefore receive the larger share of the health system benefits. Results from this study confirm that the very poor and poor as well as rural dwellers utilized PHC services the least. Policymakers and programme managers in Enugu State should design and implement payment strategies that will assure financial risk protection of the poor. There is need for the Enugu State Government to reform the health system so that it is primed for achieving UHC. In addition, the provision of health education for both urban and rural women and men will increase their knowledge on utilization of PHC services in the communities.

## Competing interests

The authors declare that they have no competing interests.

## Authors’ contributions

ILO conceived the study. ILO, OEO and FOA participated in the design of the study. FOA participated in the implementation of the study, and data analysis. ILO, OEO and FOA participated in the interpretation of results. FOA made the first draft of the manuscript. ILO and OEO commented on the paper and provided valuable guidance for manuscript write up. ILO, OEO and FOA revised the draft manuscript and all read and approved the final manuscript.

## Pre-publication history

The pre-publication history for this paper can be accessed here:

http://www.biomedcentral.com/1472-6963/14/132/prepub
